# Supplementary material for: Assemblage Structure of Ichthyoplankton Communities in the Southern Adriatic Sea (Eastern Mediterranean)
Source: Biology (Basel). 2023 Nov 19;12(11):1449. doi: 10.3390/biology12111449 (PMC10669739; doi:10.3390/biology12111449)
Supplement: Supplementary file 1 [file biology-12-01449-s001.zip › biology-2562255-supplementary.pdf]

Station	Hauls Depth Range (m)	Filtered volume (m^3)	BIONESS		Temperature (°C)	Salinity	Fluorescence (Chl *a* µg l^{-1})
			Start time	End time			
S1	90-80	40.5	20:31:00	20:38:00	12.69	38.64	0.114
	80-70	45.3	20:38:00	20:45:00	12.68	38.62	0.151
	70-60	46.4	20:45:00	20:53:00	12.82	38.64	0.371
	60-50	47.3	20:53:00	21:00:00	12.91	38.64	0.334
	50-40	66	21:00:00	21:10:00	13.22	38.65	0.957
	40-30	38.1	21:10:00	21:17:00	13.8	38.72	0.993
	30-20	42.6	21:17:00	21:24:00	14.6	38.75	0.371
	20-ott	42.9	21:24:00	21:30:00	16.42	38.31	0.517
	10-0	41.2	21:30:00	21:37:00	19.74	37.73	0.041
S3	170-160	45.2	14:12:00	14:22:00	14.16	38.82	0.041
	160-140	50.2	14:22:00	14:32:00	14.97	38.93	0.041
	140-120	50.4	14:32:00	14:42:00	14.99	38.93	0.114
	120-100	54.1	14:42:00	14:54:00	15.05	38.93	0.224
	100-80	66.3	14:54:00	15:04:00	15.19	38.93	0.371
	80-60	59	15:04:00	15:14:00	15.35	38.93	0.59
	60-40	61.6	15:14:00	15:25:00	15.75	38.92	0.151
	40-20	50.9	15:25:00	15:34:00	16.85	38.9	0.041
L41	550-400	82.1	18:38:00	18:56:00	13.58	38.74	0.004
	400-300	50.5	18:56:00	19:10:00	13.67	38.76	0.004
	300-200	69.7	19:10:00	19:22:00	13.76	38.78	0.004
	200-100	56.6	19:22:00	19:32:00	14.16	38.79	0.261
	100-80	66.3	19:32:00	19:45:00	14.68	38.82	0.371
	80-60	52.2	19:45:00	19:54:00	14.94	38.8	0.334
	60-40	45.1	19:54:00	20:03:00	15.68	38.79	0.114
	40-20	45.7	20:03:00	20:11:00	17.4	38.8	0.078
	0-20	58.3	20:11:00	20:21:00	19.54	38.74	0.114
S7	180-160	40.6	15:37:00	15:47:00	15.02	38.94	0.004

	160-140	29	15:47:00	15:54:00	15.08	38.94	0.187
	140-120	34.6	15:54:00	16:02:00	15.12	38.94	0.334
	120-100	30.1	16:02:00	16:09:00	15.18	38.93	0.48
	100-80	26.5	16:09:00	16:15:00	15.29	38.93	0.59
	80-60	35.9	16:15:00	16:23:00	15.48	38.91	0.407
	60-40	35.7	16:23:00	16:30:00	16.03	38.93	0.151
	40-20	44	16:30:00	16:38:00	17.23	38.89	0.078
	20-0	40	16:38:00	16:46:00	21.16	36.63	0.078
S20	700-600	27	15:09:00	15:18:00	13.75	38.77	0.004
	600-400	59.1	15:18:00	15:30:00	13.97	38.8	0.004
	400-300	65.3	15:30:00	15:44:00	14.1	38.83	0.004
	300-200	45.9	15:44:00	15:53:00	14.54	38.91	0.004
	200-100	52.2	15:53:00	16:05:00	14.92	38.91	0.334
	100-80	35.2	16:05:00	16:14:00	15.04	38.91	0.59
	80-60	34.3	16:14:00	16:22:00	15.24	38.87	0.81
	60-40	29.3	16:22:00	16:30:00	15.66	38.87	0.261
	40-0	43	16:30:00	16:41:00	19.1	38.83	0.041
S21	900-800	30.2	22:43:00	22:56:00	13.8	38.77	0.004
	800-600	55.1	22:56:00	23:12:00	14.08	38.84	0.004
	600-400	45.2	23:12:00	23:24:00	14.58	38.93	0.004
	400-100	65.2	23:24:00	23:44:00	15.49	38.98	0.078
	100-80	25.6	23:44:00	23:55:00	15.53	38.99	0.261
	80-60	26.7	23:55:00	00:05:00	15.51	38.96	0.59
	60-40	27.8	00:05:00	00:15:00	15.83	38.93	0.187
	40-0	29	00:15:00	00:28:00	17.61	38.85	0.114
S22	800-900	15.1	18:35:00	18:43:00	13.96	38.8	0.004
	600-800	65.1	18:43:00	19:01:00	14.2	38.85	0.004
	600-400	50.9	19:01:00	19:14:00	14.73	38.93	0.004
	200-400	46.1	19:14:00	19:24:00	15.08	38.93	0.004
	100-200	37.2	19:24:00	19:38:00	15.4	38.97	0.151
	80-100	24.7	19:38:00	19:48:00	15.45	38.97	0.444

	60-80	25.4	19:48:00	19:59:00	15.42	38.93	0.92
	40-60	31.5	19:59:00	20:10:00	15.68	38.93	0.224
	0-40	49.6	20:10:00	20:25:00	16.85	38.83	0.114
S23	1100-800	90.4	18:34:00	18:53:00	13.95	38.81	0.004
	800-600	76.6	18:53:00	19:06:00	14.33	38.9	0.004
	600-400	94	19:06:00	19:22:00	14.81	38.99	0.004
	400-200	93.5	19:22:00	19:37:00	14.99	38.93	0.004
	200-100	48	19:37:00	19:47:00	15.27	38.96	0.041
	100-80	57.4	19:47:00	19:57:00	15.42	38.93	0.59
	80-60	45.1	19:57:00	20:07:00	15.75	38.94	0.407
	60-40	47.2	20:07:00	20:17:00	16.1	38.92	0.114
	40-0	55.1	20:17:00	20:29:00	18.35	38.87	0.078
S24	1000-800	53.3	22:39:00	22:54:00	13.81	38.77	0.004
	800-600	65.8	22:54:00	23:07:00	14.07	38.84	0.004
	600-400	83.1	23:07:00	23:22:00	14.47	38.92	0.004
	400-200	74.9	23:22:00	23:34:00	14.77	38.94	0.004
	200-100	40.9	23:34:00	23:41:00	14.92	38.91	0.517
	100-80	45.8	23:41:00	23:50:00	15.09	38.91	0.883
	80-60	49.2	23:50:00	00:00:00	15.49	38.91	0.371
	60-40	51.3	00:00:00	00:10:00	16.27	38.91	0.151
	40-0	89.1	00:10:00	00:23:00	19.31	38.83	0.078
S25	210-180	33.9	04:36:00	04:46:00	14.34	38.84	0.114
	180-160	19.5	04:46:00	04:52:00	14.33	38.84	0.114
	160-140	31.6	04:52:00	05:00:00	14.34	38.84	0.187
	140-100	62.2	05:00:00	05:17:00	14.55	38.85	0.444
	100-80	32.5	05:17:00	05:25:00	14.67	38.84	0.664
	80-60	34.5	05:25:00	05:35:00	15.07	38.8	0.48
	60-40	35.4	05:35:00	05:44:00	16.04	38.98	0.151
	40-0	107	05:44:00	05:59:00	17.92	39.44	0.004
S8	310-250	69	04:59:00	05:16:00	14.93	38.94	0.004
	250-200	64.9	05:16:00	05:30:00	15.07	38.94	0.004

	200-150	43.9	05:30:00	05:40:00	15.14	38.93	0.004
	150-100	59.2	05:40:00	05:52:00	15.15	38.92	0.078
	100-80	38.5	05:52:00	06:00:00	15.23	38.93	0.297
	80-60	36.5	06:00:00	06:07:00	15.39	38.93	1.36
	60-40	37.4	06:07:00	06:15:00	15.8	38.91	0.151
	40-20	30.3	06:15:00	06:22:00	16.82	38.92	0.151
	20-0	29.3	06:22:00	06:35:00	20.21	38.73	0.151
S10	1096-800	77.3	00:26:00	00:41:00	13.63	38.77	0.004
	800-600	72.5	00:41:00	00:53:00	13.9	38.79	0.004
	600-400	79.6	00:53:00	01:05:00	13.98	38.81	0.004
	400-200	89.1	01:05:00	01:20:00	13.98	38.82	0.004
	200-100	68.7	01:20:00	01:34:00	13.99	38.82	0.444
	100-80	38.8	01:34:00	01:42:00	14.16	38.83	0.664
	80-60	45.6	01:42:00	01:51:00	14.3	38.83	0.883
	60-40	38.7	01:51:00	01:58:00	14.63	38.82	0.151
	40-0	39.9	01:58:00	02:07:00	20.16	38.8	0.078
S15	900-800	24.9	06:01:00	06:09:00	13.37	38.74	0.004
	800-600	105.4	06:09:00	06:28:00	13.62	38.76	0.004
	400-200	74.6	06:28:00	06:44:00	13.97	38.81	0.004
	200-100	49	06:53:00	07:01:00	14.59	38.88	0.151
	100-80	60.7	07:01:00	07:11:00	14.57	38.85	0.334
	80-60	47.5	07:11:00	07:19:00	14.92	38.83	0.334
	60-40	47	07:19:00	07:28:00	15.73	38.89	0.224
	40-0	57.1	07:28:00	07:38:00	19.42	38.71	0.004
S16c	300-250	30,75	11:58:00	12:05:00	14.8	38.93	0.004
	250-200	59,63	12:05:00	12:16:00	14.92	38.94	0.004
	200-150	48,00	12:16:00	12:24:00	14.94	38.92	0.004
	150-100	40,42	12:24:00	12:40:00	15.12	38.93	0.151
	100-80	40,24	12:40:00	12:47:00	15.14	38.92	0.517
	80-60	35,98	12:47:00	12:55:00	15.48	38.93	0.627
	60-40	29,86	12:55:00	12:55:00	15.79	38.92	0.151

	0-40	35,72	12:55:00	13:00:00	20.49	38.66	0.041
S19	100-90	48.1	10:23:00	10:34:00	14.68	38.88	0.261
	90-80	42.4	10:34:00	10:42:00	14.72	38.88	0.297
	80-70	49.3	10:42:00	10:51:00	14.83	38.89	0.444
	40-30	67.2	10:51:00	11:14:00	15.79	38.84	0.224
	30-20	48.3	11:14:00	11:23:00	18.76	38.42	0.297
	20-ott	25.8	11:23:00	11:31:00	18.97	38.21	0.114
	10-0	32.7	11:31:00	11:42:00	19.08	38.17	0.032
S14	600-400	57.8	18:18:00	18:33:00	13.91	38.8	0.004
	400-300	57.9	18:33:00	18:44:00	13.97	38.81	0.004
	300-200	60.4	18:44:00	18:56:00	13.94	38.78	0.078
	200-100	45.9	18:56:00	19:05:00	14.69	38.88	0.261
	100-80	34.8	19:05:00	19:14:00	14.92	38.91	0.517
	80-60	34.7	19:14:00	19:22:00	15.06	38.92	0.627
	60-40	32.6	19:22:00	19:31:00	15.29	38.89	0.371
	40-20	49.2	19:31:00	19:41:00	18.25	38.76	0.151
	20-0	46.3	19:41:00	18:51:00	18.88	38.54	0.224
S11	1060-800	47.8	07:45:00	07:56:00	13.39	38.72	0.004
	800-600	79.9	07:56:00	08:09:00	13.74	38.76	0.004
	600-400	90.2	08:09:00	08:24:00	13.97	38.81	0.004
	400-200	92.3	08:24:00	08:36:00	13.98	38.81	0.004
	200-100	62.2	08:36:00	08:46:00	14	38.82	0.114
	100-80	63.3	08:46:00	08:56:00	14.05	38.82	0.737
	80-60	63.7	08:56:00	09:06:00	14.28	38.82	0.517
	60-40	58.2	09:06:00	09:15:00	14.64	38.82	0.187
	40-0	94.7	09:15:00	09:25:00	18.41	38.8	0.032

Table S1 Sampled strata for each station along with filtered volume, time sampling, temperature, salinity and fluorescence values
